# Knowledge Evaluation of Oral Diseases and Perception of Cooperation with Dental Experts for Oral Care of Nurses in Intensive Care Units in Korea: A Preliminary Study

**DOI:** 10.3390/nursrep13010048

**Published:** 2023-03-19

**Authors:** Yesel Kim, Hye-Min Ku, Mi-Kyoung Jun

**Affiliations:** 1 Department of Dental Hygiene, Jeonju Kijeon College, Jeonju 54989, Republic of Korea; 2 Department of Preventive Dentistry & Public Oral Health, BK21 FOUR Project, Yonsei University College of Dentistry, Seoul 03722, Republic of Korea; 3 Department of Dental Hygiene, Dongnam Health University, Suwon 16328, Republic of Korea

**Keywords:** intensive care unit, nurses, oral care, oral diseases, perception

## Abstract

The aim of this study was to identify the status of education and knowledge concerning oral diseases for oral care as they relate to intensive care unit (ICU) nurses, as well as to investigate the perception of oral care education and practice, as led by dental experts. This study conducted a self-report survey consisting of 33 questions on education and knowledge about oral diseases, as well as perception of dental expert-led education and practice, targeting 240 nurses in the ICU. Finally, 227 questionnaires were analyzed, and 75.3% of the participants were staff nurses, and 41.4% were in the medical ICU. In the area of education and knowledge of major oral diseases, more than 50% of the respondents treating gingivitis, periodontitis, and dental caries did not complete dental education, and it was found that more than half of the respondents were unable to distinguish diseases of the mouth. It was recognized that more than half of nurses required dental expert-led education and practice. In this study, the education and knowledge of oral diseases of ICU nurses were found to be insufficient, and the need for the cooperation of dental experts was high. Therefore, collaboration to improve oral care practical guidelines for realistically applicable ICU patients will be needed.

## 1. Introduction

An intensive care unit (ICU) restores the lives of critically severely ill patients and minimizes organ damage, and it is a place where patients requiring intensive care and monitoring, such as medical and surgical intensive care unit patients, can be preferentially treated. Patients in the ICU have difficulty swallowing through the oral cavity, and endotracheal intubation in the ICU patients is often performed to keep their airways open. A previous study reported that oral conditions may worsen when general patients are admitted to the ICU [1]. Because the ICU patients kept their mouths open, they had a lack of saliva secretion, and this significantly reduced self-cleaning of the oral cavity. Saliva has an essential role in maintaining oral mucosa moisture, cleaning the mouth, providing an antibacterial effect, and remineralizing teeth [2,3,4]. When the saliva flow rate is reduced, the bacterial colonization of the oral biofilm on the tooth surface is accelerated, and the possibility of developing periodontal disease is increased. A reduced saliva flow can increase clinical fungal infection, which is a common opportunistic infection. This type of infection includes oral candidiasis [5].

Additionally, an important precursor for the development of ventilator associated pneumonia (VAP) is bacterial colonization of the oral cavity. The major route for acquiring VAP is oropharyngeal colonization by the oral biofilm. Patients with endotracheal intubation most likely possess routes for colonization because these same bacteria can be traced to respiratory infections, which they may have. The 2015 Centers for Disease Control and Prevention (CDC) report provides information concerning that the VAP is an important pathogenic infection occurring in the ICU patients [6]. VAP is one of the risk factors for death in ICU patients [7]. A previous study indicated that there exist the same pathogenic bacteria in VAP in the oral cavities and lungs of VAP patients [8]. This means that oral care is crucial for severely ill patients. Additionally, previous studies reported that oral care of ICU patients reduces the incidence of healthcare-associated pathogenic infections [9,10]. Meanwhile, the CDC report, which is revised and called the ‘Guideline for Prevention of Nosocomial Pneumonia’, includes chlorhexidine gluconate oral rinse in the prevention or modulation of oropharyngeal colonization [8]. These guidelines advised patients using ventilators to perform oral care with chlorhexidine daily. This treatment should be preceded by identifying the oral characteristics and dental diseases of severely ill patients for oral care, which is necessary for ICU patients. There are many studies on oral care interventions for severely ill patients, but there is a lack of studies evaluating the level of oral disease awareness among ICU nurses. Appropriate oral care for ICU patients is successful in preventing colonization by pathogenic bacteria and reducing the incidence of VAP. In a previous study that was conducted concerning a survey on the importance of nursing care for ICU nurses, oral health care was somewhat lower in priority than nursing care [11]. Oral care is not directly related to the patient’s life in an ICU, where emergency situations frequently occur. Therefore, it is difficult to actively perform oral care [12]. In a study on the attitude of nurses concerning oral care, it was reported that ICU nurses had anxiety about moving the endotracheal intubation and having it fall out in patients during oral care. Additionally, it was said that nurses were reluctant to perform oral care because oral tissue is difficult to manage, and oral care is unpleasant. In particular, patients in the ICU with bleeding tendencies are more likely to bleed due to damage to their oral mucosa during tooth brushing, which may lead to sepsis. Therefore, nurses are hesitant to actively perform oral care [13]. Evidence-based clinical nursing practice guidelines for oral care were established in 2014 in Korea [14]. This guideline suggests that ICU patients with endotracheal intubation should have oral assessments in accordance with the guidelines for high-risk patients when nurses perform oral care. It was recommended to perform oral care every 2 to 4 h using a chlorhexidine oral rinse. Despite the existence of these written guidelines, it is currently difficult for hospital wards to have oral care guidelines, and it is reported that the utilization of oral care guidelines is also low [1,15,16].

Therefore, the purpose of this study was to identify the status of oral care education completion and oral disease knowledge as it relates to intensive care unit (ICU) nurses. Additionally, we investigate the nurses’ perception of oral care education and practice, as led by dental experts. This study was conducted with the aim of describing considerations and improvements for education, as well as practical guidelines, for the oral care of ICU patients in the future.

## 2. Materials and Methods

### 2.1. Study Design

This study is a multicenter cross-sectional survey of ICU nurses. The survey was conducted online from 9 June to 20 October 2021. The study hypothesis was tested to determine the relationship between an ICU nurse’s attitude, perception, and completion of oral care education.

### 2.2. Participants and Data Collection Procedures

The participants of this study were 240 Korean ICU nurses selected through convenience sampling and were extracted from 10 general hospitals with more than 300 beds. The inclusion criteria was ICU work experience of more than 6 months, and cases with no ICU work experience or less than 6 months were excluded. The survey was requested to be distributed as a QR code and mobile link of the survey on the bulletin board of the nursing department through the nurse in charge of each hospital and was conducted as an online survey (Google form, Google Co., Mountain View, CA, USA). After the survey period was over, a certain compensation (mobile coffee coupon) was provided to the participants who submitted their consent to the survey and the answers to all questions in the survey.

### 2.3. Measurements

The survey tools used in previous studies [17] were modified and supplemented to suit the purpose of this study. The knowledge of the dental background in Korea was modified by dental experts consisting of five dental hygiene professors and dental hospital professors, and the final draft was formed after review by two ICU nurses.

The composition of the questionnaire consisted of a total of 33 questions using questionnaires, and general characteristics included 6 questions, including age, gender, level of nursing education, position, ICU work experience, and type of ICU. The questionnaire to determine the level of education and knowledge about oral diseases, the main cause of aspiration pneumonia in ICU inpatients, consisted of 24 [18,19] questions in three parts. The first part is a questionnaire about oral biofilm, dental calculus, and tongue coating consisted of 9 questions, which are the causes of oral diseases. The second part is a questionnaire about gingivitis, periodontitis, and dental caries consisted of 9 questions, which are major oral diseases. The third part included questions about oral candidiasis and dry mouth consisted of 6 questions, which are oral mucosal diseases. The final composition consisted of 3 questions about cooperation in dental expert (dentist or dental hygienists)-led oral care education and practice.

### 2.4. Statistical Analysis

Statistical analysis of the collected data was performed using the SPSS program (IBM SPSS Statistics 25.0 for window, SPSS Inc, Chicago, IL, USA). All data were organized categorically and analyzed using frequency analysis.

### 2.5. Ethical Considerations

Ethical approval for the conduct of the study was obtained from the Institutional Ethics Review Committee of Ajou University Hospital. The ethics approval number is AJIRB-SBR-SUR-21-245. All study participants were checked for consent before completing the questionnaire, and questionnaires in which they did not consent were excluded. In addition, research participants’ information obtained through survey responses was not used for any purpose other than research purposes, and information regarding anonymity and confidentiality was provided.

## 3. Results

There were 240 participants in the survey, and 227 questionnaires were finally used for the final analysis, excluding 13 questionnaires with insincere responses.

### 3.1. General Characteristics of the Participants

More than half of the participants were under the age of 30, and the gender distribution was 70.9% female and 29.1% male. As for the degree, the bachelor’s degree accounted for the majority (84.1%), and for the position, the staff nurse accounted for 75.3%. As for the type of ICU, 41.4% were in medical, 38.3% in surgical, and 20.3% in general ICU, and less than 5 years of ICU work experience was the highest at 56.4% (Table 1).

### 3.2. Completion of Education and Knowledge of the Factor of Oral Diseases

In the area of education and knowledge of the causes of oral diseases, oral biofilm showed that 93.4% of the participants did not have completion of education on oral biofilm and could not identify oral biofilm in the mouth. Oral biofilm treatment methods were also answered as ‘Don’t know’ by more than half. As for dental calculus, 54.2% of the participants did not have completion of education on dental calculus, and 44.5% answered ‘possible’ to identify dental calculus in the mouth. As a dental calculus treatment method, ‘scaling’ was the most frequent response at 49.3%. Tongue coating showed that 96.5% of the participants did not have a completion of education about tongue coating and it was ‘impossible’ to identify tongue coating in the mouth. More than half of the Tongue coating treatment methods also answered ‘Don’t know’ (Table 2).

### 3.3. Completion of Education and Knowledge of Major Oral Diseases

In the area of education and knowledge about major oral diseases, 63.9% of the participants did not have completion of education on gingivitis, and more than half of them were found to be ‘impossible’ to identify gingivitis in the mouth. Regarding the treatment method for gingivitis, ‘Don’t know’ was the most frequent response. As for periodontitis, 72.2% of the participants did not have a completion of education about periodontitis, and it was found that it was ‘impossible’ to identify periodontitis in the mouth. Regarding the treatment method of periodontitis, ‘don’t know’ was 76.2%, and ‘periodontal treatment’ was answered by only 10.2%. As for dental caries, 54.2% of the participants did not have completion of education on dental caries, and it was found that it was ‘impossible’ to identify dental caries in the mouth. As for the treatment method for dental caries, 23.8% responded ‘periodontal treatment’, and only 1.3% responded with ‘ Remove tooth decay’ (Table 3).

### 3.4. Completion of Education and Knowledge of Oral Mucosal Diseases

In the area of education and knowledge about oral mucosal disease, 55.5% of the participants had a completion of education about oral candidiasis, and 42.3% of the participants were found that it was ‘impossible’ to identify oral candidiasis in the mouth. As a treatment method for oral candidiasis, ‘application of Nystatin’ was the most frequent response at 32.2%.

As for dry mouth, 51.5% of the participants had a completion of education about dry mouth, and 49.3% were found that it was ‘impossible’ to identify dry mouth in the mouth. As for the treatment method for dry mouth, 28.6% answered ‘continuous application of wet gauze’ (Table 4).

### 3.5. Cooperation of Dental Experts and the Importance of Oral Care of ICU Patients

In the question on the need for oral care education led by dentists or dental hygienists, who are dental experts for oral care in ICU, 83.3% answered ‘need’, and 85% of oral care practices led by dentists or dental hygienists were found to be ‘Need’.

More than half (53.3%) of the responses to the importance of performing oral care among the nursing task areas recognized it as ‘important’ (Table 5).

## 4. Discussion

Oral health is closely related to general health [20]. ICU patients may not be able to manage their oral hygiene due to their systemic condition, which can lead to oral complications. Nurses should have sufficient knowledge about oral diseases and properly perform oral care for severely ill patients.

### 4.1. ICU Nurses’ Knowledge Conditions of Dental Disease and Prevention

This study confirmed that ICU nurses were less awareness of prevention of dental disease and dental treatment. It was 93.4% of ICU nurses who did not complete oral care education. However, it was confirmed that ICU curses responded that oral care led by dental experts was necessary. In particular, ICU nurses had a low understanding of dental plaque, dental calculus, and tongue plaque, which are representative causes of dental diseases. Oral care of patients admitted to the ICU is easily overlooked in terms of importance, and it is reported that there is a large deviation in nurses’ knowledge and practical skills [21]. The Absence of oral care performed by nurses can affect the deterioration of oral health in ICU patients. It has been confirmed that patients in ICU are more likely to develop oral health problems as medication taken during hospitalization may decrease the amount of saliva secretion [22,23]. Previous study reported that patients in ICU have a decreased oral health practice. Dental plaque is attached to tooth surfaces and then dental caries and gingival bleeding occurs. And it has been confirmed that patients in ICU have a high rate of oral xerostomia [24]. Another previous study reported that nurses in ICU did not assess the oral health of inpatients and could neglect oral care of inpatients because there was no oral care guideline [25]. In Korea, evidence-based clinical nursing practice guidelines were developed in 2014, but many medical institutions did not have these guidelines in wards [16]. Also, the utilization of practical guidelines was reported to be low which is similar to other countries. In particular, patients in ICU with poor systematic conditions can develop oral diseases rapidly due to reduced salivary secretion. Promptly eliminating local factors cause oral disease that could prevent the development into other complications. Therefore, it is believed that video education using application for nurses on the initial cause of oral disease is necessary.

The response rate of ICU nurses who did not receive training on how to treat gingivitis and periodontitis caused by plaque and calculus was high. However, nurses’ education and understanding of oral candidiasis, which is a disease of the oral mucosa, were high. Half of the ICU nurses included in the study had experience with xerostomia education. It is believed that this is because basic oral care is being performed as a method to prevent Ventilator Associated Pneumonia (VAP) in the major hospital ICU. According to a report by the American Association of Critical Care Nurses in 2007, oropharyngeal plaque colonization is a major risk factor for VAP, so intensive management is required and plaque can be removed by tooth brushing [26]. It is necessary to manage plaque by using chlorhexidine among chemical mouthwashes in addition to mechanical cleaning such as tooth brushing. It is proposed to lightly massage the lips and oral mucosa using the moisture gel [27].

### 4.2. Education Completion of Oral Care Program for ICU Nurses

Meanwhile, 30% of the nurses included in this study responded that they knew how to treat oral candidiasis and xerostomia. ICU patients generally take a lot of medication. Most drugs cause dry mouth as a side effect [28]. Oral xerostomia serves as a risk factor for the development of oral candidiasis. The lack of saliva reduces the ability to resist oral candidiasis because saliva contains antifungal enzymes. Patients in ICU may undergo steroid therapy using ventilator for a long time and antibiotic therapy may cause changes in the patient’s immune function [29]. It has been reported that there is a potential benefit of prophylactic antibiotics of antifungal agents to prevent fungal infection from occurring in patients who have been taking high-dose corticosteroids for a long period of time rather than treating oral candidiasis infection after it develops [29]. Infectious Diseases Society of America (IDSA) guidelines recommended that Fluconazole, an antifungal agent effective in preventing fungal infection in patients with cancer treatment, is recommended for use in moderate to severe oropharyngeal candidiasis [30]. In particular, oral care is essential to prevent xerostomia and oral candidiasis in ICU patients, and it is necessary to remove odors and stimulate saliva secretion. Therefore, ICU nurses should recognize that oral health can be associated with the systemic health of intensive care patients. I suggest that nurses will recognize the importance of oral care when oral care is included as essential education completion.

In this study, 83% of nurses responded that it was necessary to the questions of “whether or not dental professionals-led oral care education is necessary for oral care.” However, half of the nurses answered that the importance of oral care in nursing care area was not important. This means that the oral care is relatively unimportant in the ICU nursing care, and it is believed that it is not directly related to patient life. In the future, even if the practical oral care is established and the oral care is carried out under the leadership of dental experts, the experts in the two fields can cooperate only when the ICU nurses can accurately assess the oral condition of the ICU patient. It is suggested that nurses needs to complete education on oral disease-related knowledge and training in prevention methods. Previous study has also confirmed that the more education nurses receive, the higher their performance, so it is important to provide oral care education to nurse [31]. It will be necessary for nurses to recognize the requirement of oral care for patients in ICU and to improve the practice guidelines so that they can be usefully used in clinical practice based on evidence-based practice guidelines for oral care.

### 4.3. Limitations

This study is a survey study of ICU nurses in some hospitals in one country. In the future, it is suggested that it is necessary to evaluate oral nursing knowledge for multilateral nurses.

Finally, an oral care system should be introduced in accordance with national policies to provide continuous and systematic oral management for ICU patients. In addition, oral health experts need constant efforts to develop and expand various intervention programs for ICU nurses.

## 5. Conclusions

This study confirmed that the education and knowledge of oral diseases of ICU nurses were found to be insufficient. However, ICU nurses were aware of the need for oral care cooperation with dental professionals. It is necessary to develop practical oral care guidelines for ICU nurses. ICU nurses should be trained in oral care and accurately judge patients’ current oral conditions. They have to communicate the patient’s condition to dental professionals when patients with severe oral conditions, and then provide appropriate oral care to the patient.

## Figures and Tables

**Table 1:** General characteristics of icu nurses in Korea (N = 227).

Variable	N	%
Age (years)		
<30	149	65.6
30–39	56	24.7
≥40	22	9.7
Gender		
Male	66	29.1
Female	161	70.9
Level of nursing education		
Bachelor’s degree	191	84.1
≥Master’s degree	36	15.9
Position		
Staff nurse	171	75.3
Charge or Head nurse	56	24.7
Type of ICU		
Medical	94	41.4
Surgical	87	38.3
General	46	20.3
Experience working in ICU (years)		
<5	128	56.4
5–9	62	27.3
≥10	37	16.3

Abbreviation: ICU, intensive care unit.

**Table 2:** Completion of education and knowledge of the factor of oral diseases (N = 227).

Variable	N	%
Completion of education on oral biofilm		
No	212	93.4
Yes	15	6.6
Oral biofilm identification in the mouth of the patient		
Impossible	88	36.8
possible	5	2.2
Don’t know oral biofilm	134	59.0
Oral biofilm treatment method		
Mouthwash gargle	2	0.9
Toothpaste and brushing	11	4.8
Mouthwash and brushing	10	4.4
Scaling	3	1.3
Don’t know	201	88.6
Completion of education on dental calculus		
No	123	54.2
Yes	104	45.8
Dental calculus identification in the mouth of the patient		
Impossible	66	29.1
Possible	101	44.5
Don’t know dental calculus	60	26.4
Dental calculus treatment method		
Water gargle	2	0.9
Mouthwash gargle	2	0.9
Toothpaste and brushing	11	4.8
Mouthwash and brushing	7	3.1
Scaling	112	49.3
Periodontal treatment	4	1.8
Don’t know	89	39.2
Completion of education on tongue coating		
No	219	96.5
Yes	8	3.5
Tongue coating identification in the mouth of the patient		
Impossible	88	38.8
Possible	9	4.0
Don’t know tongue coating	130	57.3
Tongue coating treatment method		
Water gargle	2	0.9
Mouthwash gargle	4	1.8
Toothpaste and brushing	10	4.4
Mouthwash and brushing	18	7.9
Scaling	1	0.4
Don’t know	192	84.6

**Table 3:** Completion of education and knowledge of major oral diseases (N = 227).

Variable	N	%
Completion of education on gingivitis		
No	145	63.9
Yes	82	36.1
Gingivitis identification in the mouth of the patient		
Impossible	130	57.3
possible	25	11.0
Don’t know gingivitis	72	31.7
Gingivitis treatment method		
Water gargle	3	1.3
Mouthwash gargle	3	1.3
Toothpaste and brushing	5	2.2
Mouthwash and brushing	10	4.4
Scaling	4	1.8
Periodontal treatment	23	10.1
Antibiotic	15	6.6
NSAIDs	2	0.9
Don’t know	162	71.4
Completion of education on periodontitis		
No	165	72.7
Yes	62	27.3
Periodontitis identification in the mouth of the patient		
Impossible	117	51.5
Possible	18	7.9
Don’t know periodontitis		40.5
Periodontitis treatment method		
Mouthwash gargle	2	0.9
Toothpaste and brushing	6	2.6
Mouthwash and brushing	7	3.1
Scaling	1	0.4
Periodontal treatment	23	10.1
Antibiotic	14	6.2
NSAIDs	1	0.4
Don’t know	173	76.2
Completion of education on dental caries		
No	123	54.2
Yes	104	45.8
Dental caries identification in the mouth of the patient		
Impossible	85	37.4
Possible	81	35.7
Don’t know dental caries	61	26.9
Dental caries treatment method		
Water gargle	2	0.9
Mouthwash gargle	2	0.9
Toothpaste and brushing	15	6.6
Mouthwash and brushing	12	5.3
Scaling	2	0.9
Periodontal treatment	54	23.8
Antibiotic	3	1.3
Remove tooth decay	3	1.3
Don’t know	134	59.0

Abbreviation: NSAIDs, Non-steroidal anti-inflammatory drugs.

**Table 4:** Completion of education and knowledge of oral mucosal diseases (N = 227).

Variable	N	%
Completion of education on oral candidiasis		
No	101	44.5
Yes	126	55.5
Oral candidiasis identification in the mouth of the patient		
Impossible	77	33.9
possible	96	42.3
Don’t know oral candidiasis	54	23.8
Oral candidiasis treatment method		
Water gargle	1	0.4
Mouthwash gargle	6	2.6
Toothpaste and brushing	4	1.8
Mouthwash and brushing	8	3.5
Antibiotic	29	12.8
NSAIDs	1	0.4
Nystatin	73	32.2
Triamcinolone Acetonide Ingredient Ointment	2	0.9
Naturopathy	1	0.4
Don’t know	102	44.9
Completion of education on dry mouth		
No	110	48.5
Yes	117	51.5
Dry mouth identification in the mouth of the patient		
Impossible	58	25.6
Possible	112	49.3
Don’t know dry mouth	57	25.1
Dry mouth treatment method		
Water gargle	24	10.8
Mouthwash gargle	23	10.1
Toothpaste and brushing	2	0.9
Mouthwash and brushing	7	3.1
Continuous wet gauze	65	28.6
Continuous chlorhexidine gauze	5	2.2
Antibiotic	7	3.1
Humectant	2	0.9
Water intake	1	0.4
Don’t know	95	40.1

**Table 5:** Cooperation of dental experts and the importance of oral care of ICU patients (N = 227).

Variable	N	%
Oral care education led by dental experts (dentist or dental hygienists)		
Need	189	83.3
Unnecessary	38	16.7
Oral care practice led by dental experts (dentist or dental hygienists)		
Need	193	85.0
Unnecessary	34	15.0
Importance of oral care among nursing task areas		
Unimportant	16	6.2
Neutral	90	39.6
Important	121	53.3

## Data Availability

Not applicable.
